# Effects of Death Anxiety Experienced by Individuals with Myocardial Infarction: A Phenomenological Approach

**DOI:** 10.3390/healthcare14142054

**Published:** 2026-07-08

**Authors:** Fatma Buruntekin, Özlem Ceyhan

**Affiliations:** 1 Institute of Health Sciences, Erciyes University, Kayseri 38039, Turkey; fburuntekin@gmail.com; 2 Faculty of Health Sciences, Department of Internal Diseases Nursing, Erciyes University, Kayseri 38039, Turkey

**Keywords:** death anxiety, myocardial infarction, phenomenology, lived experience, psychological impact, chronic disease, coping, qualitative research, patient experience, mental health

## Abstract

**Aim:** This study sought to investigate in depth the experiences of individuals who had a myocardial infarction (MI), focusing on death anxiety and life changes. Design: A qualitative phenomenological design was employed. **Methods:** Criterion-based purposive sampling was used. Data were collected between June and July 2023 through semi-structured interviews. Seven participants were interviewed, and six were included in the analysis. Data were analyzed using content analysis. **Results:** Four main categories emerged: (1) experiences of patients, (2) changes in daily life, (3) thoughts about death, and (4) recommendations. Participants reported intense fear of death, lifestyle changes, and increased awareness of mortality. **Conclusions:** Death anxiety is a prominent psychological response after MI that significantly affects patients’ perceptions and behaviors. Psychosocial interventions are recommended to help patients cope with these experiences.

## 1. Introduction

Chronic diseases, also known as non-communicable diseases (NCDs), have become a global health issue in recent years. These diseases, including cardiovascular diseases, cancer, chronic respiratory diseases, and diabetes, have far-reaching consequences for individuals, communities, and health systems. According to the World Health Organization (WHO), chronic diseases account for approximately 71% of all global deaths, with cardiovascular diseases (CVDs) alone accounting for more than 17 million deaths each year. The burden of chronic diseases goes beyond mortality, with individuals experiencing long-term disability, reduced quality of life, and increased health costs [1,2,3,4,5].

CVDs, a group of chronic diseases, encompass a range of conditions affecting the heart and blood vessels, including coronary artery disease, heart attacks, stroke, heart failure, and hypertension. These diseases are a global health problem and continue to be the leading cause of morbidity and mortality worldwide [5,6,7]. Myocardial infarction, commonly known as a heart attack, is a critical and life-threatening condition that leads to the death of part of the heart muscle as a result of a severe reduction or blockage of blood flow to the heart muscle [8,9]. Myocardial infarction is one of the leading causes of death worldwide and can lead to serious complications such as cardiac arrest, arrhythmias, heart failure, and even sudden death [10,11].

The experience of living with a chronic illness and the possibility of life-threatening events such as a heart attack or stroke can trigger thoughts and worries about death. Studies have determined that individuals with cardiovascular disease, such as a heart attack or stroke, are more likely to experience death anxiety than those without these conditions. However, these studies have also reported that anxiety causes more cardiovascular disease [12,13,14].

Death anxiety is a multidimensional psychological and existential construct referring to distress, fear, or apprehension associated with awareness of mortality and the inevitability of death [15,16]. Although it may overlap with general anxiety, depressive symptoms, or trauma-related fear, death anxiety specifically involves concerns about non-existence, loss of self, and existential meaning. Unlike generalized anxiety disorders characterized by diffuse and persistent worry across various life domains, death anxiety is directly linked to mortality awareness and existential threat [17]. Similarly, while depressive cognition often involves hopelessness and negative self-appraisal, death anxiety is primarily centered on existential fear and the anticipated end of personal existence. From an existential perspective, awareness of death is considered a fundamental aspect of human consciousness that may provoke anxiety but also stimulate meaning-making processes [16]. In individuals experiencing life-threatening conditions such as myocardial infarction, death anxiety may therefore emerge not only as a psychological response but also as an existential confrontation with mortality.

Studies have provided qualitative evidence that individuals’ experiences after myocardial infarction (MI) can affect their lifestyles, their perceptions of their bodies, and the healthcare system. Moreover, it has been stated that individuals need support after discharge. On the other hand, it was also reported that individuals did not receive any support or received inadequate support [18,19,20].

Experiencing a heart attack can significantly alter one’s life, both physically and emotionally. It often encourages individuals to reflect on their mortality and reassess their priorities and values [16,21,22]. Irvin Yalom sees death anxiety as a fundamental aspect of human existence. He believes that awareness of our mortality is a natural part of being human. He argues that this awareness influences our thoughts, feelings, and behaviors. In his therapeutic approach, Yalom emphasizes that death anxiety can affect individuals in various ways, such as shaping their choices, beliefs, and behaviors. According to Yalom, individuals should be encouraged to confront and explore their death anxiety as a means of personal growth and self-awareness. By facing their mortality, individuals can gain a deeper understanding of themselves, their values, and the choices they make in life [16,21].

In addition to physical consequences, myocardial infarction has significant psychological effects, including anxiety, depression, and, particularly, death anxiety. Previous studies have demonstrated that individuals with cardiovascular diseases report higher levels of death anxiety compared to healthy populations. Researchers have discovered that factors such as age, gender, prior psychological status, and perceived social support influence the intensity of death anxiety. For instance, younger patients may experience greater existential distress, while individuals with limited social support may report higher anxiety levels. Moreover, psychological constructs such as coping style, resilience, and illness perception play a crucial role in shaping patients’ responses to MI. Despite these findings, in-depth qualitative exploration of death anxiety in MI patients remains limited [13,18,19].

Recent empirical studies have demonstrated that death anxiety is highly prevalent among patients following myocardial infarction. For instance, research comparing patients with acute myocardial infarction, cancer patients, and healthy individuals has shown that individuals with myocardial infarction report significantly higher levels of death anxiety than other groups and that these levels are associated with sociodemographic variables such as age, gender, education, and employment status [23].

In addition, psychological factors such as depression, general anxiety, coping strategies, and perceived social support have been identified as important determinants of death anxiety in this population. Higher levels of anxiety and depressive symptoms are associated with increased mortality risk and poorer psychological adjustment following myocardial infarction [24]. Younger patients and individuals with lower social support tend to experience more intense existential distress, while adaptive coping mechanisms may reduce the psychological burden.

Despite these findings, most existing studies are quantitative in nature and focus on symptom levels rather than the subjective meaning and lived experience of death anxiety. Therefore, a qualitative exploration is needed to understand better how patients interpret and integrate this experience into their lives.

Fear of death after a heart attack can significantly affect a person’s mental and emotional health, leading to depression and reduced quality of life. It can also hinder the overall recovery process by affecting motivation, participation in necessary lifestyle changes, and adherence to treatment plans [25,26,27]. Emphasizing the importance of a healthy and satisfying life after MI is crucial to eliminating the fear of death. Individuals can shift their focus from the fear of death to maximizing their post-MI life by concentrating on the present moment and establishing realistic future goals [27]. Yalom emphasizes that death anxiety is not necessarily pathological, but rather a natural outcome that can motivate individuals to seek meaning, purpose, and fulfillment in life. Excessive death anxiety can disrupt daily functioning and result in psychological disorders [16,21]. By examining the experiences of individuals who have had myocardial infarction, the present study will guide patients to reduce their anxiety and raise awareness of those who will help these patients (family members, health professionals, etc.). In line with this purpose, the study sought to answer the research question “What are the thoughts of individuals about death anxiety after myocardial infarction?”.

## 2. The Study

### Aim

This study aimed to explore the lived experiences of individuals after myocardial infarction, with a particular focus on the nature, meaning, and impact of death anxiety and related life changes.

## 3. Methods

### 3.1. Design

Phenomenology is a philosophical approach that focuses on the study of conscious experience and how individuals perceive, interpret, and understand the world around them. It seeks to understand the essence and meaning of subjective experiences without relying on external influences or preconceived assumptions. Researchers aim to uncover the basic structures and meanings underlying individual experiences, allowing for a deeper understanding of human consciousness and subjective reality [28,29]. In this study, the thoughts of individuals with myocardial infarction about death anxiety were investigated in depth, and their experiences were tried to be revealed from a qualitative perspective. For all these reasons, phenomenology, one of the qualitative research method designs, was used in the study.

### 3.2. Sampling and Recruitment

In the study, a criterion-based study group, one of the purposeful sampling types, was used. In criterion sampling, a type of purposive sampling, researchers define criteria or characteristics that participants must meet to be included in the study. These criteria can be based on various factors such as age, gender, profession, experience, expertise, or specific characteristics related to the research topic [29,30].

Although death anxiety is not classified as a diagnostic entity in DSM-5-TR, the experiences reported in this study may overlap with several DSM-5-TR-related constructs, including anxiety disorders, adjustment disorder, acute stress reactions, depressive symptoms, and illness anxiety disorder. However, the aim of this study was not to establish psychiatric diagnoses, but to explore lived experiences qualitatively within a phenomenological framework.

In this study, no structured psychiatric diagnostic interview or formal psychological screening instrument was administered. Participants were included based on their medical diagnosis of myocardial infarction and willingness to participate. Individuals were not excluded based on the presence of self-reported psychological symptoms, as the focus was on subjective lived experience rather than psychiatric classification. These factors should be considered when interpreting the findings.

A small sample size was considered appropriate in accordance with the principles of phenomenological research, which prioritize depth and richness of lived experience rather than statistical generalization. In phenomenological research, sample size is determined by the depth and richness of participants’ experiences rather than statistical representativeness. Creswell and Poth (2018) indicate that phenomenological studies commonly involve a small number of participants, often ranging from approximately 3 to 10 individuals [31]. Data collection continued until sufficient depth of information was achieved, and no new themes emerged across interviews.

Participants were recruited from the cardiology service of a public hospital in Turkey. The study was conducted in Turkey because the researchers aimed to explore the lived experiences of myocardial infarction survivors within the specific sociocultural and healthcare context of Turkey. Criterion-based purposive sampling was used. Inclusion criteria were (1) being diagnosed with myocardial infarction at least one year prior, (2) being over 18 years of age, and (3) willingness to participate. Interviews were conducted in Turkish by the principal researcher in a private setting within the hospital. Each interview lasted approximately 25–30 min and was audio-recorded with participant consent. Transcriptions were performed verbatim in Turkish, and no translation was required during analysis. Seven participants were initially interviewed; however, one interview was excluded from the final analysis due to incomplete and insufficient responses that did not allow for meaningful thematic interpretation. Data saturation was considered achieved when no new themes emerged from the interviews. After six participants, response repetition indicated saturation.

### 3.3. Demographic Characteristics

A summary:

Participants (*n* = 6) consisted of adult individuals diagnosed with MI. The sample included both male and female participants with varying ages and backgrounds. Most participants reported lifestyle changes following MI, including quitting smoking, reduced physical activity, and increased health awareness.

Table 1 presents a detailed overview of the participants’ demographic and clinical characteristics, enhancing the transferability of the findings.

Clinical characteristics included time since myocardial infarction, ongoing treatment status, and self-reported psychiatric history. This information was included to improve the contextual understanding and transferability of the findings.

### 3.4. Data Collection

An interview is a qualitative research data collection tool that involves a direct conversation between a researcher and a participant, or interviewee. It is a structured or semi-structured process of interaction that aims to gather in-depth information, perspectives, and insights about a particular research topic or phenomenon. Interviews allow for a comprehensive exploration of participants’ experiences, beliefs, attitudes, and perspectives on the research topic [29,30,31,32]. The study utilized a semi-structured interview form and a personal information form developed by the researcher. The responsible researcher conducted the interviews face-to-face. A semi-structured interview form consisting of seven questions and probes for the first and second questions was created in line with the literature [18,19]. The interview form was presented to three experts, two from the Faculty of Health Sciences and one from the Faculty of Education. The third and fifth questions were removed from the interview form with the expert’s recommendation because they overlapped with the previous questions.

Interviews were conducted by the principal researcher with seven patients with myocardial infarction who were being treated at a public hospital in June–July 2023 at convenient times. Data collection took six weeks. Before starting the interviews, the principal researcher introduced herself and stated that participants’ answers to the interview questions would remain confidential and would not be used for any purpose apart from this study. A total of 7 participants were interviewed, but 1 was excluded because his responses were not suitable for the study, and sufficient data could not be obtained. The interviews lasted approximately 25–30 min. On the day the data were collected, the recordings were transferred to a Word document and presented to participants to prevent data loss. The research was terminated when the data started to repeat each other in line with the qualitative research method. The final version of the semi-structured interview guide, including the main interview questions and probing prompts, is presented in Appendix A. Demographic and clinical information collected from participants is presented in Appendix B. The semi-structured interview guide was developed by the researchers based on the relevant literature and the study objectives. The guide was reviewed by experts to evaluate the clarity, relevance, and comprehensiveness of the questions. Minor revisions were made based on expert feedback before data collection.

Reflexivity was maintained throughout the research process. The primary researcher, a nurse with experience in qualitative research, acknowledged her preconceptions regarding myocardial infarction and psychological responses. To minimize bias, reflective notes were kept during data collection and analysis, and the researcher continuously bracketed personal assumptions to remain close to participants’ lived experiences.

The present study adopted a phenomenological design and an inductive content analysis approach. Accordingly, the interview questions were intentionally designed to elicit participants’ lived experiences rather than to assess predefined theoretical constructs or variables. Therefore, the interview guide was informed by the existing literature on myocardial infarction experiences, death anxiety, and post-myocardial infarction adaptation and was developed to encourage participants to describe their experiences in their own words. Following expert review, the interview guide was refined for clarity and relevance before data collection.

### 3.5. Data Analysis

The research data were analyzed by content analysis. The essence of content analysis is to organize and interpret similar data clearly by grouping them under codes and themes [29]. In the analysis, the stages reported by Creswell (2009) [28] were used.

Based on the data obtained, separate codes were created for each question. Categories and themes were created in line with the resulting codes. The codes are presented under each category in the tables in the Findings Section. In the analysis of the study data, direct quotations were used to obtain detailed data. Although the study was designed within a phenomenological framework, data were analyzed using an inductive content analysis approach. This decision was made because content analysis allows for the systematic identification of patterns, meanings, and categories derived directly from participants’ narratives while remaining aligned with the phenomenological aims of exploring lived experience. The focus of the analysis was not merely to describe the frequency of codes but to interpret the underlying meanings embedded in participants’ experiences of death anxiety following myocardial infarction. Themes and categories identified in the study:

Theme: Myocardial infarction.

Categories:

- Experiences of patients with myocardial infarction;
- Changes in patients’ lives after myocardial infarction;
- Patients’ thoughts about death after myocardial infarction;
- Recommendations for patients with myocardial infarction.

The research team evaluated data saturation when patients began repeating the same answers during data collection. Two researchers who evaluated the codes and themes determined that data saturation had been achieved and that data collection was complete with 6 participants.

### 3.6. Ethical Considerations

All necessary permissions were obtained for the study. Ethical approval for the study was obtained from the Nevşehir University Rectorate Non-Interventional Clinical Research Publication Ethics Committee, with decision number 2023/15. The study was conducted in accordance with the principles of the Declaration of Helsinki. All participants provided written informed consent before participating and were informed that they could withdraw at any time without any consequences. All identifying information was removed from the interview records, and only the codes were used in the final analysis.

### 3.7. Validity–Reliability

In qualitative studies, researchers are expected to rigorously test the internal validity and reliability of the study model and data collection tools and report and present them to readers [29].

To ensure credibility, member checking was conducted by presenting transcribed data to participants for confirmation. Detailed descriptions of the participants, the setting, and the research procedures supported transferability. During the interview, the participant’s answers were confirmed, and the transcribed data were then presented to the participant for approval. In addition, the study was presented to two experts, one from the Faculty of Health Sciences and the other from the Faculty of Education, from beginning to end. Dependability and confirmability were ensured through independent coding by two researchers and expert review. Discrepancies were discussed until consensus was reached.

To ensure the external validity (transferability) of the study, the research model, data collection tool, process, participants, and data analysis section were described in detail. Furthermore, purposive sampling in selecting the study group was considered an important factor in enhancing external validity.

To ensure the study’s internal reliability (consistency), the findings section was presented without comment, and only direct quotations were used. Moreover, both researchers reached a consensus on the codes created. All codes could be agreed upon.

To ensure the external reliability (confirmability) of the study, it was submitted to the same experts for review, who confirmed that the Findings, Analysis, Conclusion, and Discussion Sections were harmonious and mutually reinforcing.

## 4. Findings

In this section, the findings from patients’ experiences with myocardial infarction are presented. Findings are presented in categories in each table. Moreover, codes are presented according to the participants in the tables.

As seen in Table 2, in line with the answers received from the participants, it is seen that there are eight different thoughts in the category of experiences of patients with myocardial infarction regarding anxiety. Five participants stated that they experienced pain during the disease. One of these patients (P1) stated, “*I had a lot of pain. It was like a knife was stabbed in my heart, chest, back, and shoulder. I came to the hospital immediately. I thought I was going to die. God forbid that kind of pain.*”, while P4 said, “*I felt pain near the heart. I couldn’t make sense of it at first.*”

Four participants stated that they experienced fear of death during the disease. One of these patients (P2) stated, “*They immediately took me to angiography. It was as if I went to the other side. My heart rhythm was disturbed, they massaged me and gave me a shock. It was as if a truck ran over me.*” P3 stated, “*I was scared in the hospital when they said heart attack. A great fear gripped me, then I said that this heart attack would kill me.*” P5 said, “I was scared, I immediately informed my relatives, I was alone in the hospital…”

Two participants reported sweating. One of these patients, P5, stated, “*I had a crisis once in the mosque, I sweated a lot. After that, we came to the hospital with my son immediately.*” P2, who stated that he experienced difficulty in breathing, stenocardia, and arm numbness, said, “*The first time, I was not aware that I had a heart attack. I had arm numbness, and my back hurt. In my next heart attack, my heart started to squeeze while I was driving the car, my chest hurt, and I had difficulty breathing.*” P6, who experienced fatigue and a burning sensation in the throat, stated, “*I walked 100 meters, but I felt tired as if I had run 10 km. I was out of breath. I had a burning in my throat.*”

As shown in Table 3, there are 7 statements in the category of changes in patients’ daily lives after myocardial infarction. Three participants stated that they quit their jobs after having a heart attack. One of these participants, P2, stated, “There was a change in my work life. Now I don’t work as much as before,” while P3 stated, “*When I had my first heart attack, I didn’t go to work for a month, then I returned to work. I worked for 4 years, then I quit my job.*”

Two participants stated that they quit smoking after MI and felt tired. One of these participants, P5, said, “*I quit smoking… I got tired, that’s why I quit… I had an angioplasty 15–16 months ago. I have no more energy.*” P6, another participant who quit smoking, stated that he started taking regular medication and quit sports, “*Since that day, I started taking medication more regularly. I take medication all the time. I also quit smoking. I was playing football, but I quit. I don’t do sports that require effort. Apart from that, nothing has changed.*”

One participant (P1) stated that he could not get out of bed for a long time, “*I lay in bed for a year, I did not do any work. I could not get up, so I slept at my daughter-in-law’s house.*” One participant (P4) stated that there was no change in his daily life, “*There was no change.*”

As seen in Table 4, in line with the answers received from the participants, it is seen that there are five different thoughts in the category of death-related thoughts of patients after myocardial infarction. Three participants stated that they constantly thought about death after the disease. One of these patients, P2 stated, “*Before I had a heart attack, I never thought about death. Now I can’t stop thinking about it. It is always with me.*”, while P3 said, “*In the past, when I was young, death did not come to my mind, I used to say I would live long and travel…. But now I don’t know if I will live tomorrow….*”.

Two participants stated that they felt closer to death after myocardial infarction. One of these participants (P1) stated, “*When I was young, I did not think that I would die, but now I do.*” Another participant, P6, said, “*Death was very far away before. Now it seems a little closer*…” P3 expressed the thought that he was prepared for death after myocardial infarction, “*I used to not think about death. I was unprepared before, but now I am prepared.*” P5, on the other hand, stated that he realized that life had an end after the disease and there was an afterlife, “*When I had a heart attack, it never occurred to me that life would have an end… Real life will begin after this world… This thought did not occur to me before.*”

As shown in Table 5, there are 8 statements in the recommendations category for patients with myocardial infarction, consistent with the participants’ responses. Two participants stated that health should be protected. One of these participants (P1) stated, “*Everyone’s health is their own business. Those who do not take care of their health will suffer…*” Moreover, P4 stated, “*I advise older people to protect their health. Everyone should protect their health.*”

Two of the participants who had MI suggested enjoying life. One of these participants (P2) stated, “*I recommend people to enjoy life… Now I am on a diet, so I cannot eat or drink everything. Let people eat what they want when they are healthy.*” Another participant, P3, who suggested enjoying life, said, “*Everyone should live their lives while they are young… They should travel a lot.*”

One participant stated that smoking should not be practiced as a recommendation. P4 stated that people should not smoke, “*My advice to everyone is to quit smoking and take care of your health. I had health problems because of smoking. Smoking is very harmful to people.*” One participant stated that people should not be exhausted. P2 stated, “*People should not overwork and be exhausted.*”

Another participant, P5, who had MI, suggested consulting a doctor immediately in the presence of severe chest pain, “*If you have severe pain in your arms, back, and chest, go to the hospital immediately. So, they should have an angiogram immediately. I tell everyone to go immediately, have an angiogram, and don’t lose a minute. I ruined my heart because I did not have an angiogram. If I had had an angiogram, my heart would not have been so damaged.*”

Another patient suggested eating a healthy diet, having general health checks, and avoiding stress. P6 said, “*…nutrition is very important. People should pay attention to their nutrition and have periodic tests. My triglycerides were very high for 10 years. I had a friend who had high triglycerides. He was on medication, but I had never taken any medication; he is better now. There is also the stress factor. You need to stay away from stress.*”

## 5. Discussion

It was concluded that the participants had experiences of pain, fear of death, sweating, difficulty breathing, stenocardia, arm numbness, fatigue, and burning sensations in the throat related to myocardial infarction. Participants shared their physical and psychological experiences from different perspectives. The statements that the participants used for physical experiences such as pain, difficulty breathing, and arm numbness, which are symptoms of a heart attack, such as “*In my next heart attack, my heart started to squeeze while I was driving the car, my chest hurt and I had difficulty breathing and I had a crisis once in the mosque, I sweated a lot. After that, we came to the hospital with my son immediately.*” show that they used these experiences as a comparison tool for another coronary disease that may occur in the future. The statements “*I was scared, I immediately informed my relatives, I was alone in the hospital…*” used by the participants for psychological experiences such as fear of death indicate that the person’s life is actually under threat and that they are concerned that their life is coming to an end. The literature supports these participants’ physical and psychological experiences. For instance, Brännström et al. (2006) described the meaning of living with heart disease as being aware that one is going to die soon on the one hand and wanting to survive life-threatening conditions on the other [33]. Iles-Smith et al. (2017) reported that participants frequently experienced fatigue and weakness associated with myocardial infarction symptoms and linked these to symptoms they might experience in the future [34]. Therefore, these studies are considered important because they will increase awareness about early diagnosis by interpreting MI symptoms [34].

In the present study, it was concluded that myocardial infarction caused changes in participants’ daily lives, such as quitting work, quitting smoking, being unable to get out of bed for a long time, and stopping sports. The statements “*I don’t work anymore*”, “*I quit smoking*”, and “*I lay in bed for a year*” that the participants used for these changes showed they can affect the daily lives of individuals. Condon and McCarthy (2006) stated that the participants wanted to change their lifestyles because negative lifestyles, such as smoking, stress, and an unhealthy diet, lead to MI [35]. This study determined that change can occur in both directions. On the one hand, the changes experienced in daily life are positive lifestyle changes, such as quitting smoking; on the other hand, they are negative changes that limit physical activity, such as quitting work or staying in bed all the time. The lack of information is thought to cause a bidirectional change in daily life after MI, and reducing patients’ death anxiety can help. Therefore, the conclusion drawn from this study, as reflected in the participants’ opinions, is that interventions such as education and public service are needed to encourage positive lifestyle changes in patients after myocardial infarction [36,37].

Building on these findings, the present study further explores the psychological meaning of death anxiety. The findings of this study indicate that death anxiety is a central psychological experience following myocardial infarction [22]. Consistent with the existing literature, participants reported fear of death, increased awareness of mortality, and emotional distress. However, this study extends previous research by revealing the subjective meanings attributed to these experiences. Participants described death anxiety not only as fear but also as a transformative experience influencing life priorities, behaviors, and coping strategies. This aligns with existential theory, particularly Yalom’s perspective that awareness of death can lead to personal growth. Rather than claiming a transcendental essence in the strict phenomenological sense, the study aimed to capture the shared patterns and core meanings embedded within participants’ lived experiences of myocardial infarction and death anxiety. These shared meanings represent a convergence of experiences rather than an abstract phenomenological essence. Another important finding is the bidirectional nature of lifestyle changes. While some participants adopted healthier behaviors such as quitting smoking, others experienced negative consequences such as withdrawal from daily activities. This highlights the complex psychological impact of MI.

It was observed that the participants had thoughts about dying after the disease, feeling that they were close to death, being prepared for death, realizing that life has an end, and being held accountable in the afterlife. The expression, “… *I can’t stop thinking about death. It is always with me.*” “*Now it seems a little closer*”, “*…Now I am prepared.*” “… *When I had a heart attack, it never occurred to me that life would have an end… The real life will begin after this world*” shows that the person accepts the end of his existence in this world, but clings to the afterlife with his religious beliefs. According to Yalom, death means the end of personal existence [16]. The thought of the end of this existence can be deeply disturbing. The fear of extinction can create anxiety about losing one’s identity, consciousness, and connection with the world. Şahan et al. (2018) reported that patients with MI had higher death anxiety than patients with cancer or healthy individuals [23]. Although descriptive studies on death anxiety in patients with MI have been reported in the literature, this study provides in-depth evidence of the anxiety patients experience [23].

Based on their experiences, the participants in the study suggested that people should take care of their health, enjoy life, avoid smoking, avoid exhaustion, consult a doctor immediately if they have severe chest pain, eat a healthy diet, have regular health checks, and avoid stress. The statements, “*Everyone should protect their health*”, “*Enjoy life*”, “*Quit smoking*”, “*People should not overwork and be exhausted*”, “*If you see severe chest pain, go to the hospital immediately*” and “*People should pay attention to their nutrition and have their tests done once in a while… You need to stay away from stress.*” indicate that they want to provide spiritual guidance to healthy individuals by explaining the negative experience of disease and the subsequent feeling of death anxiety. Colori (2022) stated that sharing the experience helps others recognize the seriousness of the patient’s pain and be inspired to recover [38]. At the same time, it makes the patients feel that they are not alone [38]. Ziebland and Wyke (2012) reported that reading people’s descriptions of their experiences is more memorable because they provide living evidence [39]. Hence, knowing that others are dealing with similar problems and wanting to guide them may reduce the feelings of loneliness and anxiety of MI patients. At the same time, this study is important because it provides evidence presented by patients with MI to healthy people.

The present study was not designed to evaluate an intervention, predict clinical outcomes, or determine factors associated with reducing myocardial infarction recurrence or mortality. Rather, its purpose was to explore the lived experiences of individuals following myocardial infarction and to understand the meanings they attributed to these experiences. Accordingly, the value of the four categories lies in improving understanding of patients’ psychological, emotional, and existential experiences after myocardial infarction. These findings may assist healthcare professionals in recognizing patients’ concerns, identifying areas requiring psychosocial support, and developing more patient-centered educational and counseling approaches. Given the exploratory phenomenological design, the findings should be interpreted as providing an in-depth understanding of participants’ lived experiences rather than evidence of causal relationships or clinical effectiveness.

## 6. Limitations

This study has several limitations. First, the sample size is small, which may limit the generalizability of the findings; however this limitation is consistent with the phenomenological research tradition, which prioritizes depth over breadth. Second, the study was conducted in a single center, which may limit the transferability of the results to other settings. Third, the use of self-reported data may introduce recall bias. Additionally, as in all qualitative interviews, the interaction between the researcher and participants may have influenced the responses obtained. Furthermore, participants’ interpretations of death anxiety may have been influenced by the cultural and religious context in which the study was conducted, which may limit transferability to different cultural settings.

## 7. Future Directions

Future studies should include larger and more diverse samples to explore demographic differences in death anxiety. Longitudinal research is recommended to examine changes over time. Additionally, intervention-based studies focusing on reducing death anxiety in MI patients would be valuable. Future studies should also explore the influence of cultural, religious, and psychosocial factors on death anxiety using multi-center and cross-cultural qualitative designs.

## 8. Conclusions

This study demonstrates that death anxiety is a significant and multidimensional experience among individuals who have experienced myocardial infarction. Beyond fear, it shapes patients’ perceptions, behaviors, and life choices.

The findings highlight the importance of integrating psychological and emotional support into post-MI care. Healthcare professionals should consider death anxiety as a critical component of recovery and develop interventions aimed at improving patients’ psychological well-being.

## Figures and Tables

**Table 1:** Participant Characteristics.

Participant	Age	Gender	Marital Status	Education	Time Since the First MI	Treatment Status	Psychiatric History
P1	80	Female	Widowed	Non-literate	3 years	Completed	No
P2	49	Male	Married	Primary school	8 years	Completed	No
P3	59	Male	Married	Primary school	15 years	Completed	No
P4	75	Female	Married	Primary school	3 years	Completed	No
P5	65	Male	Married	Primary school	11 years	Completed	No
P6	54	Male	Married	University	4 years	Completed	No

**Table 2:** Experiences of Patients with Myocardial Infarction.

Codes	Total Incidence (*n*)	Number of Participants
Pain	5	P1, P2, P3, P4, P5
Fear of death	4	P2, P3, P5, P6
Sweating	2	P3, P5
Difficulty breathing	2	P2, P6
Stenocardia	1	P2
Arm numbness	1	P2
Fatigue	1	P6
Burning sensation in the throat	1	P6

**Table 3:** Changes in the Daily Life of Patients After Myocardial Infarction.

Codes	Total Incidence (*n*)	Number of Participants
Quitting work	3	P1, P2, P3
Quitting smoking	2	P5, P6
Inability to get out of bed for a long time	1	P1
No difference	1	P4
Feeling tired	1	P5
Start taking regular medication.	1	P6
Quitting sports	1	P6

**Table 4:** Death-Related Thoughts of Patients After Myocardial Infarction.

Codes	Total Incidence (*n*)	Number of Participants
Constantly thinking about death.	3	P2, P3, P4
Feeling close to death	2	P1, P6
Preparedness for death	1	P3
Realizing that life has an end	1	P5
Thinking about the afterlife	1	P5

**Table 5:** Recommendations by Patients with Myocardial Infarction.

Codes	Total Incidence (*n*)	Number of Participants
Attention to health	2	P1, P4
Enjoying life	2	P2, P3
No smoking	2	P4, P6
No getting exhausted	1	P2
Urgent medical attention is required in the presence of severe chest pain	1	P5
Healthy nutrition	1	P6
General health checks	1	P6
Avoiding stress	1	P6

## Data Availability

The data that support the findings of this study are available from the corresponding author upon reasonable request. This is because the data is not publicly available due to privacy concerns and ethical restrictions.

## Appendix A. Semi-Structured Interview Guide

Introduction Participants were informed about the purpose of the study, the voluntary nature of participation, confidentiality procedures, and audio recording of the interview. Interviews lasted approximately 20–30 min. Main Interview Questions 1. What was it like for you to experience a myocardial infarction? Probe: ○ How did experiencing a myocardial infarction make you feel? 2. What changes did you experience in your daily life following this experience? Probes: ○ Changes in work life ○ Changes in family life ○ Changes in social life 3. How did this experience affect your thoughts about death? 4. Based on your experiences with myocardial infarction, what recommendations would you like to give to others? 5. Is there anything else you would like to add that I have not asked about?

## Appendix B. Demographic and Clinical Information Form

Participants were asked to provide the following demographic and clinical information: 1. Age: ___________ 2. Gender: (1) Female (2) Male 3. Marital status: (1) Married (2) Single 4. Education level: (1) Illiterate (2) Primary education (3) High school (4) University and above 5. Occupation: (1) Retired (2) Civil servant (3) Self-employed (4) Other: ___________ 6. Employment status: (1) Employed (2) Unemployed 7. Place of residence: (1) City (2) District (3) Town (4) Village 8. Time since first myocardial infarction diagnosis: ___________ 9. Current medication use related to myocardial infarction: (1) Yes—please specify: ___________ (2) No 10. Presence of any chronic disease: Please specify: ___________ 11. History of psychiatric illness (diagnosed or treated): (1) Yes (2) No (If yes, please specify): ___________
